# The Impact of Hot Ambient Temperature and Prolonged Fasting Duration during Ramadan on Patients with Chronic Kidney Disease: A Literature Review

**DOI:** 10.1155/2023/2636507

**Published:** 2023-12-09

**Authors:** Ali AlSahow

**Affiliations:** Division of Nephrology, Jahra Hospital, Jahra, Kuwait

## Abstract

The Islamic (lunar) calendar has 11 fewer days each year than the Gregorian (solar) calendar. Consequently, ambient temperatures during the month of Ramadan and the duration of the presunrise-to-sunset fast will change each year. At some point, individuals observing Ramadan will experience prolonged periods of fasting during the hot summer months. In this manuscript, findings published in the English-language medical literature that address the impact of prolonged fasting during the warmer summer months on patients with chronic kidney disease, including dialysis and transplantation patients, are reviewed. This is of particular concern given the accelerated pace of global warming. The limitations of the evidence that is currently available are also discussed, and an approach that might be used to standardize future evaluations of the impact of fasting on kidney health is suggested.

## 1. Introduction

The Islamic year is defined by a lunar calendar and includes 354 days and is thus 11 days shorter than the Gregorian calendar year. Therefore, the month of Ramadan begins 11 days earlier each year relative to the Gregorian calendar and occurs in a completely different season every nine years (i.e., a full rotation through all four seasons every 33 years). Consequently, the daily duration of the fast and ambient temperatures during the month of Ramadan changes not only according to geographic location but also over time. This seasonal shift may require participants to experience prolonged fasting during the hot summer months.

## 2. The Significance of Fasting Duration

Muslims worldwide are expected to follow the sunrise and sunset times at their location to mark the beginning and end of the daily Ramadan fast. This requirement is maintained even during the summer, when the daily fast may exceed 16 hours in the northernmost regions. However, if prolonged daily fasting makes it difficult to perform one's job or may harm one's health, it is permissible to break the fast and resume it at any time before the next Ramadan by fasting for the same number of days as those missed [1, 2]. Some scholars, however, have suggested that Muslims residing in locations that would require fasting for more than 20 hours might begin their daily fast according to local sunrise time and fast for the same number of hours as would be required of those currently living in Mecca; in other words, individuals in these extreme geographical locations might cease fasting even if sunset has yet to occur [3]. This duration variability is one of the confounding variables that precludes an accurate assessment of the effects of Ramadan fasting on health-related biomarkers [4]. Long fasting duration may have an indirect effect on kidney health, including disruptions of sleep and circadian rhythm because of the sudden shifts in meal times and disruption of the timing and frequency of medications [5, 6].

## 3. The Significance of a Hot Ambient Temperature

### 3.1. Heat Stress

Heat stress is the sum of the heat generated by the body (e.g., clothing and physical activities) and the heat generated by the environment (e.g., air temperature, radiant temperature, humidity, and air velocity), minus heat lost from the body to the environment through dry and evaporative loss (e.g., perspiration) [7–10]. It is influenced by age, sex, body surface area, body mass index (BMI), heat/exercise acclimatization, preexisting comorbidities (e.g., cardiovascular disease (CVD), diabetes mellitus (DM)), alcohol intake, medications (antipsychotics, sedatives, diuretics, laxatives, anticholinergics, beta-blockers, calcium channel blockers), and hydration status [7–10]. It is also influenced by occupational heat gain from working in hot weather (e.g., agricultural work during the summer, working near furnaces and boilers), physical activity (e.g., construction), protective clothing (e.g., firefighting), [11, 12], and housing conditions (e.g., homes with poor ventilation and no air conditioning) [9]. Heat stress that overwhelms the physiological thermoregulatory compensatory mechanisms can elevate core body temperature (i.e., heat strain) and lead to heat-related illnesses (HRIs) that can be categorized as minor (e.g., edema, cramps, rash), mild (e.g., heat syncope), moderate (e.g., heat exhaustion), or severe (e.g., heat stroke with or without multiorgan failure) [9]. Wet bulb globe temperature (WBGT) is frequently used to predict the likelihood of HRI. This tool measures heat stress in direct sunlight and takes into account temperature, humidity, wind speed, sun angle, and cloud cover (i.e., solar radiation) [13]. A WBGT measurement above 32.0°C indicates a high risk of HRI among those with physically demanding outdoor jobs as well as patients in vulnerable groups [14].

### 3.2. Global Warming, the Kidney, and Ramadan fasting

The climate is warming at an unprecedented rate, as the global surface temperature was 1.09°C higher in 2011–2020 than in 1850–1900. As a consequence, heat extremes are increasing in frequency, duration, and intensity [15]. Temperature is rising in all parts of the world, especially the tropics and the Middle East-North Africa (MENA) regions, where most of the Muslim majority countries are. These regions are already warm and dry, and now, with global warming, they are becoming worse. A rise in temperature above 2°C will also increase the risk of floods, wildfires, and draught [16–20]. Global warming will definitely increase the risks of HRIs and impact kidney health while fasting Ramadan during the long, hot summer months as a result of the increased incidence of serious kidney-related problems brought about by global warming. These problems include heat-induced fluid loss from perspiration, which can lead to reduced urine volumes and increased urine supersaturation of stone-forming salts [21–23] in susceptible individuals, most notably those presenting with predisposing genetics and socioeconomic factors (i.e., diet, area of residency, occupation, and exposure to sunlight) [24]. This fluid loss is not replaced while fasting, compounding the situation. It also includes acute kidney injury (AKI), which can result from rhabdomyolysis induced by extreme heat exposure and/or from hypovolemia [21–23]. Recurrent episodes of AKI can lead to chronic kidney disease (CKD) and eventually kidney failure. Furthermore, patients with CKD are at increased risk of future episodes of AKI [25–28]. Global warming is projected to cause floods that disrupt water treatment facilities, thereby reducing water supply and resulting in water contamination [29]. Water contamination will increase the prevalence of water- and vector-borne diarrheal diseases associated with poor hygiene and sanitation in low- and middle-income countries (LMICs) and lead to dehydration, hypovolemia, and AKI [23, 30]. At the same time, prevention strategies designed to prevent vector-borne diseases are frequently cost-prohibitive in these locations [23]. This is highly relevant given the possibility that Ramadan fasting in extremely hot weather for long hours may increase the risk of AKI in CKD patients, and rising temperatures and water shortages will only make the situation worse, especially in LMICs where infrastructure for early detection and management of AKI is already limited. A rise in the incidence of recurrent urinary tract infections with reduced urine volume, especially in people with poor hydration habits and poor hygiene, is another expected problem with global warming [22, 23, 25]. In addition, global warming could further increase the prevalence of CKD of unknown etiology or chronic interstitial nephritis in agricultural communities (CINAC), which is a condition that primarily affects agricultural workers in warmer LMICs, that may result in part from recurrent and persistent exposure to occupational heat stress and dehydration, which is exacerbated by prolonged fasting in hot weather [21–23].

## 4. Impact of the Duration of Fasting and Hot Ambient Temperatures on Kidney Health

### 4.1. Any Evidence?

Full papers on Ramadan and kidney health published in English-language databases listed in PubMed, Scopus, Web of Science, and Google Scholar databases that focused on the impact of Ramadan on kidney health were reviewed for evidence of the impact of prolonged fasting and high ambient temperatures on patients with kidney disease. Fasting duration for the date (when reported) and location of each study was obtained from reference [31], and ambient temperature during Ramadan at the time each study was performed was obtained from references [32, 33]. The studies reviewed were divided into four groups, including patients with or without kidney disease, nephrolithiasis, dialysis patients, and kidney transplant recipients. Methods and outcomes are summarized in Tables 1–4 and Tables 5–8, respectively.

#### 4.1.1. Impact of Fasting on Kidney Function in Study Subjects with or without Nondialysis (ND) CKD

Twenty-five studies that addressed kidney function in these patient cohorts were identified and of these studies, only one was a multicenter study, two were retrospective, and one failed to report the statistical analysis method used. Two of the 25 studies were conducted during the winter months, 21 were conducted during the summer, and one did not report the date of the study. The methods used and outcomes from these studies are summarized in Tables 1 and 5, respectively.

One of the aforementioned retrospective studies was conducted in Turkey and compared fasting CKD patients who developed AKI during the months of Ramadan in 2013 and 2014 to fasting CKD patients who did not develop AKI. While high blood pressure (BP) and the number of days that each study participant fasted were significant risk factors for AKI, treatment with renin-angiotensin-aldosterone system inhibitors (RAASi) was not. CKD patients who developed AKI tended to be older with higher baseline levels of creatinine and proteinuria. Study participants were evaluated only during Ramadan; no estimated glomerular filtration rate (eGFR) values were provided before, during, or after this month [34].

The second retrospective study was performed in the Kingdom of Saudi Arabia (KSA), where the authors attempted to determine the incidence of AKI during the months of Ramadan (assuming the likelihood of fasting) compared to Shawal (the following month) in 2016 and 2017. The results revealed a statistically significant higher incidence of AKI during Shawal, with high BP, a history of AKI, and concomitant cirrhosis as significant predictors. The authors concluded that the higher incidence of AKI might be attributed to higher ambient temperatures in Shawal than in Ramadan during the years included in this study [35].

Three prospective studies that did not include nonfasting control groups evaluated the development of worsening kidney function (WKF) and AKI. Results from a study conducted in Morocco revealed that 12% of the participants developed AKI (one at Stage 1–2, five at Stage 3, and one at Stage 4). Five participants recovered completely while two recovered only partially. No specific risk factors were identified except for a baseline creatinine clearance (Cr Cl) <60 mL/min. While the average baseline (pre-Ramadan) Cr Cl for all study participants was 72.9 mL/min, Cr Cl values during and after Ramadan were not reported [36]. Another prospective study that was conducted in Turkey evaluated the incidence of AKI Stage 2 just before Iftar (the postsunset meal). The results of this study revealed a small (5 *μ*mol/L) but statistically significant increase in mean creatinine levels just before Iftar that did not meet acute kidney injury network (AKIN) criteria (i.e., thirst period <48 hours long and creatinine increase <26.5 *μ*mol/L) [37]. The third study from the KSA evaluated fasting CKD-ND (Stages 3–5) and reported WKF in 34% of the 36 participants, including 15 during and seven after Ramadan, with 7/36, 12/24, and 3/5 with stages 3, 4, and 5 disease, respectively. Of note, eight participants improved. Lower baseline eGFR, higher baseline systolic BP, and younger age were reported as risk factors for WKF. There was no effect on serum potassium. However, no Ramadan or post-Ramadan eGFR values were provided [38]. One undated study without a control group reported a significant decline in Cr Cl from pre-Ramadan baseline of 17.2 mL/min during Ramadan that persisted for two weeks [39].

One study performed in Egypt that was conducted during the winter used technetium (Tc)-99m stannous diethylene-triamine-pentaacetate (DTPA) renography to measure GFR before and during Ramadan in patients with CKD. While they observed no significant changes in GFR, the authors reported a small increase in urinary N-acetyl-beta-D-glucosaminidase (NAG), suggesting renal tubular injury, and significantly higher potassium levels during Ramadan [40]. A study conducted in the United Arab Emirates (UAE) included estimated Cr Cl values collected before, during the final week, and one month after Ramadan. Among their findings, the authors reported statistically significant improvements in eGFR during and after Ramadan, with no risk of hyperkalemia and no changes in the urine protein-creatinine ratio (PCR) [41]. However, a study focused on fasting diabetics in Egypt conducted during the summer reported a significant increase in urinary albumin-creatinine ratios (ACRs) from baseline but did not discuss kidney function [42].

The search identified six prospective studies that were conducted in the summer that included nonfasting control groups. The results of one prospective study from Turkey that focused on patients with polycystic kidney disease (PCKD) revealed no significant changes in eGFR, potassium levels, 24-hour urine volumes, or urine biomarkers. However, the authors did report significant reductions in proteinuria in the fasting group [43]. Another study from Egypt reported a decline in eGFR during Ramadan, especially among patients treated with RAASi and diuretics; the authors also noted an increased risk of cardiovascular (CV) events in patients with preexisting CV disease (CVD) and early increases in creatinine levels. Interestingly, creatinine levels were not significantly different from those reported at baseline three months after Ramadan [44]. Results from a third study conducted in Turkey revealed no significant changes in pre- and post-Ramadan eGFR measurements between the two groups with a trend towards improved eGFRs among those who were fasting. Of note, the four patients in each group that experienced WKF were older and undergoing treatment with diuretics [45]. Another study conducted in Turkey revealed that although 12.5% of fasting and 7.5% of nonfasting patients developed WKF, this difference did not achieve statistical significance. However, the authors reported that DM and proteinuria were both risk factors for WKF [46]. The authors of a study from the United Kingdom (UK) reported no significant changes when comparing pre and postRamadan creatinine levels, PCRs, weight, or BP either within or between the two groups, and no episodes of AKI [47]. Results from the final study in this group conducted in Turkey reported a small but statistically significant increase in eGFR among patients in the fasting group and a decline in eGFR among those who were not fasting, with no effects on ACR or potassium levels [48].

The remaining prospective, uncontrolled studies evaluated changes in eGFR or Cr Cl. The authors of one study conducted in the UAE reported no significant changes in eGFR, weight, BP, or potassium levels [49]. Similarly, results from a study conducted in Egypt revealed significant changes in creatinine levels, weight, BMI, muscle and bone mass, total body water, and visceral fat using electrical bioimpedance after Ramadan fasting, accompanied by an insignificant rise in eGFR [50]. Similarly, results from a study conducted in India revealed no significant differences in mean creatinine and eGFR before *versus* after Ramadan fasting. Four patients with CKD-ND (Stages 4–5) exhibited WKF; two of these patients improved after Ramadan while two did not. Low eGFR was the only risk factor associated with WKF [51]. One study conducted in Egypt split fasting diabetics into three groups: Group 1, ACR >30 and 60 mL/min/1.73 m^2^ < eGFR <90 mL/min/1.73 m^2^; Group 2, ACR >30 and eGFR >90 mL/min/1.73 m^2^; and Group 3, no CKD. ACR increased and eGFR decreased significantly from pre-Ramadan to post-Ramadan in groups 2 and 3 but not in group 1 [52]. Another study from Egypt revealed a small but statistically significant increase in mean creatinine and mean urine ACR after Ramadan; no eGFR values were reported [53]. The results of a study conducted in Singapore revealed statistically significant reductions in weight, BP, and eGFR during Ramadan from values obtained at baseline. Increases in serum potassium levels observed during Ramadan correlated with reductions in eGFR reduction but not with RAASi use [54]. Similarly, a study from Pakistan reported a significant decline in the mean eGFR six weeks after Ramadan. However, there were no changes in the mean eGFR in the 29 patients with a mean baseline eGFR of 72 mL/min/1.73 m^2^ who presented for follow-up at 12 months. The number of patients in this study with eGFRs <60 mL/min/1.73 m^2^ was not reported; however, reductions in eGFR were the same in both CKD and nonCKD patients [55]. A study from Morocco reported a significant decline in eGFR together with an increase in hydration (as measured by bioimpedance) and a 67% increase in creatinine levels. While the risk of AKI was significantly higher in patients with an eGFR <60 mL/min/1.73 m^2^, it is not clear how many of the patients enrolled in this study were in this category. The authors did not comment on any changes in the ACR [56]. Another study from Egypt reported a significant increase in Cr Cl during Ramadan among nondiabetic patients on vitamin E. All patients also experienced nonsignificant reductions in ACR; precise values for Cr and Cl were not reported [57]. A prospective study from KSA that evaluated patients with CKD-ND (Stages 3–5) reported no significant changes in eGFR, urine volume, or proteinuria [58].

#### 4.1.2. Risk of Nephrolithiasis with Fasting

Of the eight studies reviewed on this topic (four prospective and four retrospective), none reported eGFR. Two multicenter and three retrospective studies compared the incidence of nephrolithiasis during Ramadan to that reported for other months. Three of the studies took place during the summer, two were conducted during the cooler months, and one evaluated the incidence of nephrolithiasis in the summer compared to the winter. Three of the studies (two prospective and one retrospective) recruited males only. None of the retrospective studies confirmed fasting status. The methods used and outcomes from these studies are summarized in Tables 2 and 6, respectively.

Two prospective studies reported the results of a urinalysis only; both of these studies recruited males only and did not report dates. While one study reported no significant reductions in urine volume [59], another highlighted significant reductions in urine volume during Ramadan with no increased risk of calculus formation [60].

The six remaining studies reported the incidence of renal colic in Ramadan compared to all other months. A retrospective study from KSA compared the incidence of renal colic during Ramadan in winter *versus* summer as well as in non-Ramadan summer and winter months over a five-year period. The findings revealed that Ramadan fasting did not increase the risk of urinary calculi. This was the only study in which nephrolithiasis was confirmed with a computed tomography scan [61]. A prospective study from Iran conducted during the summer reported a higher incidence of renal colic during the first two weeks of Ramadan compared to periods before, during the last two weeks during, or after Ramadan. The authors concluded that ambient temperature did not contribute to these findings [62]. A retrospective study from Bahrain reported a higher incidence of renal colic during Ramadan and the month thereafter than during the preceding month. Unfortunately, the effects of ambient temperature were not evaluated [63]. The other two studies reported a higher incidence of renal colic in warmer months and no increased incidence in Ramadan; however, Ramadan was observed during the cooler months at that time [64, 65]. A prospective study from Pakistan reported *s* similar incidence of renal colic during Ramadan and the following month (Shawal), despite the warmer weather experienced during Ramadan [66].

#### 4.1.3. Ramadan Fasting and Dialysis

There were nine studies (eight prospective and four multicenter) that focused on Ramadan fasting by hemodialysis (HD) patients. The methods used and outcomes from these studies are summarized in Tables 3 and 7, respectively. One retrospective single-center study considered HD-associated mortality over 24 years (1989–2012). Among their findings, deaths were most frequent (with respect to the Gregorian calendar) in December and January (i.e., the cooler months in the Northern Hemisphere). Similarly, the largest number of deaths with respect to the Islamic calendar took place during Ramadan, which was observed in the cooler months during most of the study period; this effect was not observed in the preceding (Shaban) or the following (Shawal) months. The authors suggested that changes in dietary habits were a contributing factor to the overall higher rate of mortality during Ramadan. The fasting status of the deceased patients was not reported [67].

Four of the prospective studies included a nonfasting control group, and three were conducted during the summer months [68–70]. One was a multicenter study that evaluated patients before and during Ramadan but did not report the statistical analysis method used. This study reported no significant changes in interdialytic weight gain (IDWG), BP, or potassium levels, but did note an increased number of missing sessions among those in the fasting group [68]. The other two studies compared the outcome of daily fasting to fasting on-and-off HD days and no fasting whatsoever. A single-center study conducted in Palestine evaluated patients before, during, and after Ramadan and reported higher IDWG and potassium levels among those in the daily fasting group and only slightly higher IDWG for the partial fasting group, most notably among the patients diagnosed with diabetes [69]. By contrast, an Egyptian multicenter study evaluated patients before and after Ramadan only and reported small but statistically significant reductions in BP overall and changes in potassium levels in two of the groups. No changes in the urea reduction ratio were observed among members of the fasting group, and IDWG was not reported [70]. Another single-center prospective study that did not include dates reported no increase in IDWG or potassium levels [71]. Three additional prospective studies had no nonfasting control groups and were also conducted during the summer.

Two multicenter studies from Malaysia were reviewed. The first reported no change in dry weight, IDWG, BP, or potassium levels when comparing pre-Ramadan values to those measured at the end of Ramadan [72]. The second study combined patients who were fasting more than 20 days and those fasting fewer than 20 days (22% partial) and reported significant reductions in IDWG in regular fasting patients (especially among the females) and significant (albeit small) increases in potassium and decreases in phosphorus levels [73]. In addition, one single-center study from KSA that evaluated patients before, during, and after Ramadan reported no change in Kt/V, IDWG, BP, or potassium, despite significant reductions in the duration of each HD session [58]. A prospective single-center study from KSA that did not report the date of study or the sex of subjects evaluated patients before and during Ramadan and reported small but significant increases in IDWG and potassium levels; however, none of these changes led to visits to the emergency department [74].

Only one study assessed the feasibility of Ramadan fasting for patients maintained on peritoneal dialysis (PD) [75]. This was a comparatively small (18 patients only), single-center, prospective study conducted at the end of the summer (average temperature of 35°C and average fasting duration of 14 hours) with no nonfasting control group. The treatment schedules for both continuous ambulatory peritoneal dialysis (CAPD) and continuous cycler peritoneal dialysis (CCPD) were modified to avoid exchanges during fasting hours. Patients were evaluated pre-Ramadan, every two weeks during Ramadan, and four weeks post-Ramadan. Occupations and physical activity were not reported. Although three patients had to break their fasts, the results revealed no significant change in Cr Cl, Kt/V, or urine output between pre- and post-Ramadan values.

#### 4.1.4. Impact of Fasting on Kidney Transplant Recipients

Twelve studies that focused on the impact of Ramadan fasting on kidney transplant recipients were identified. All but one of these studies were prospective in nature, and all but one were performed at a single center. All 12 studies collected pre-, during-, and post-Ramadan values, and six of these studies included a nonfasting control group. Two studies did not report the statistical analysis methods used [76, 77], and another did not report the date of the study [78]. Only two studies were conducted during the warmer months. The methods used and outcomes from these studies are summarized in Tables 4 and 8, respectively. Overall, and despite differences in baseline values, these studies reported no statistically significant change in eGFR [79, 80]. The studies that were conducted in the cooler months were all prospective and included three from KSA: two without a control group [81, 82] and one with a control group; [83] one from Algeria without a control group; [84] one from Kuwait with a control group and was the largest; [85] one from UAE without a control group; [86] and one from Iran with a matched control group [87]. All these studies showed that fasting in cooler months was safe for kidney transplant recipients. There was also a meta-analysis of eight studies that evaluated the overall impact of Ramadan fasting on kidney transplant recipients between 1999 and 2019 and included 549 patients from five Middle East/North Africa (MENA) countries. Only two of the studies included in this meta-analysis were conducted during the summer; both of these studies included a nonfasting control group. Results from five studies with 442 fasting patients that served as their own controls revealed no significant changes in eGFR after Ramadan. While three studies that used nonfasting patients as controls reported a significant decline in eGFR, the authors of the meta-analysis noted that this finding was associated with significant publication bias [88].

## 5. Limitations of the Current Medical Literature

The evidence extracted from the aforementioned reviewed studies is limited and provides no specific guidelines or recommendations for Ramadan fasting in individuals living with kidney diseases for a variety of reasons. Given the obligatory nature of Ramadan fasting, it is not possible to perform randomized-controlled interventional trials. Individuals who have been deemed fit to fast must do so and cannot be randomized to a nonfasting group. Thus, all published studies on Ramadan fasting are by their nature noninterventional and observational. While most of these studies were prospective, several were retrospective, and most were small and performed at a single center. In addition, most studies had the fasting subjects serving as their own controls, and only a few studies had a nonfasting control group. Some studies evaluated the incidence of AKI during Ramadan compared to other months; however, this is not a true nonfasting control group not only because of the different dates and weather conditions but also because of the daily rituals and other dietary habits associated with Ramadan no longer being followed. The mean age of participants was <60 years in all of the kidney transplant and nephrolithiasis studies. Only one hemodialysis (HD) study and eight of the 25 studies that compared healthy and CKD patients featured a mean age of 60 years or more. While some studies assessed changes in eGFR from a measured baseline, others compared the incidence of WKF or AKI using the Kidney Disease: Improving Global Outcome (KDIGO) definition of the latter condition [89]. Studies used different methods to calculate eGFR [90, 91]. Some used the chronic kidney disease-epidemiology collaboration formula (CKD-Epi) or the modification of diet in renal disease formula (MDRD). Similarly, while some studies used the Cockcroft-Gault (CG) formula to determine Cr Cl, others only measured creatinine levels. One study evaluated renal function using a DTPA scan [40]. Different methods were used to assess the impact of Ramadan fasting on patients with kidney disease. Some assessed variables pre-, during-, and post-Ramadan, while others focused on pre- and during Ramadan or during- and post-Ramadan only. The timing of the post-Ramadan observations also differed between studies. Studies designed to evaluate Ramadan fasting in HD patients focused either on changes in Kt/V or IDWG. It is also important to point out that it is difficult to obtain blood samples from fasting HD patients before Iftar. Most of these patients can be evaluated only after Iftar, when they present for an HD session. The Iftar meal would certainly alter the blood biochemistry profile, patient weight, and BP measurements obtained. Although all studies aimed to assess the impact of fasting for the entire month of Ramadan, there are frequently numerous interruptions (for traveling, acute illness, or menstruation). Thus, many individuals do not fast for the entire month. This may have a profound influence on the outcomes of these studies. The number of missed days was reported differently in each study (e.g., partial or complete fast, fasting >25 days or <20 days, and so on), and many did not report when the interruption occurred (i.e., in early, middle, or late Ramadan). Some HD studies reported that participants fasted only on non-HD days. Many of the CKD studies suffered from poor patient adherence to scheduled follow-up appointments. Some studies excluded CKD patients who were deemed unfit to fast. However, it was not clear whether or not these excluded patients actually fasted. If they did, the outcomes should have been reported as real-life evidence. It is also important to mention that there is only one single-center prospective study on the outcomes of Ramadan fasting among patients undergoing PD [75].

### 5.1. Disparity of Results from Published Literature

The considerable disparity in results obtained in studies with similar methods and objectives is the result of a variety of reasons that includes differences in the timing of Ramadan and the study location. These factors will, by their nature, have a profound impact on ambient temperature, humidity, and the duration of the day-long fast. The temperature and fasting duration can differ dramatically depending on whether Ramadan falls in the summer or the winter in a given location and year. These factors may have an impact on dietary intake, meal frequency, and physical activity. Countries situated near the equator experience no significant temperature variability between months. Of note, some studies did not report the year in which the study was conducted. It also includes differences in the timing of the blood sampling during fasting hours. Timing can vary from early morning to just before Iftar and might even be performed after Iftar in the case of HD patients. Some studies did not report the timing of blood collection. Most of the studies also lacked accurate data on subjects' dietary habits, physical activity, occupation, and/or exposure to weather. One or more of these variables may influence the outcome of the Ramadan fast. For example, several Islamic countries, including the KSA, UAE, Kuwait, and Qatar, have instituted changes to working hours during the month of Ramadan. Many of the retrospective studies assumed that the recruited subjects were fasting during Ramadan. Another important factor is air-conditioning. while air conditioning is widely available in the Arabian Gulf states, this is not the case in many other parts of the world. This may alter the duration of exposure to hot weather and hence the degree of volume loss, particularly during the warmest summer months.

### 5.2. Suggested Standards for Data Reporting

Researchers in this field need to develop a universally accepted definition of high average temperature (e.g., above 35°C) and prolonged fasting duration (e.g., more than 16 hours). All reports on the impact of fasting during Ramadan need to include the location, the dates of study, the average temperature during the study days, the average fasting duration, the number of days fasting, and the statistical methods used to evaluate the findings. They should also include participant age, sex, occupation (e.g., outdoor *versus* indoor, physical *versus* sedentary work), living conditions (e.g., living alone or in a group/family setting), type of diet, and availability of air conditioning. Medications, particularly the frequency of diuretic use and adherence to amounts prescribed, should be reported. It is desirable to have a control group of individuals who are not fasting during Ramadan. Changes in eGFR should be calculated using the CKD-Epi formula as suggested by KDIGO [92] using pre-Ramadan, Ramadan, and post-Ramadan values. Timing for the pre- and postvalues should also be standardized (for example, four weeks before and four weeks after the fast). AKI should be diagnosed as per the KDIGO definition and staged based on pre-Ramadan, Ramadan, and post-Ramadan values. The timing for the pre- and postvalues should be standardized, as noted above. Assessments of HD patients should include changes in mean IDWG and mean Kt/V from pre-Ramadan values. Patients may be instructed to weigh themselves at home before mealtime to determine accurate values for IDWG. Changes in BP or signs of postural hypotension, weight gain, lower limb edema, and/or dyspnea from pre-Ramadan findings should be noted throughout the study period. Symptoms such as lethargy, anorexia, nausea, or headache that suggest electrolyte disturbances or WKF should be reported. The standardization of medical research on Ramadan fasting was originally suggested in 2015. Unfortunately, this has not yet been fully embraced by researchers in the field [93].

## 6. Conclusions

Ramadan fasting during the prolonged daylight hours and high ambient temperatures experienced during the summer months might be expected to be harmful to the health of patients with kidney disease. Unfortunately, the literature on this subject remains limited, because of the reasons discussed above and provides no clear insight on this subject. Thus, it is difficult to evaluate the individual effects of each of these two variables, particularly in patients with eGFRs <45 mL/min/1.73 m^2^, in patients older than 65 years of age, and when participants' occupations and exposures to the environment have not been considered. The Muslim community as a whole would benefit from larger studies (e.g., multicenter and multinational) that use standardized methods similar to those suggested above. While Ramadan will be observed during the winter and early spring months in the Northern Hemisphere during the next few years, these issues will reemerge once it is once again observed during the summer, particularly given the predictions for future worsening global warming.

## Figures and Tables

**Table 1:** Methods of studies on impact of Ramadan fasting on subjects with or without chronic kidney disease.

Reference	Type of study	Number of centers	Nonfasting controls (Y/N)	Sex (M/F)	Average age (years)	Dates^*∗*^ (summer: 5/15–9/15)	Fasting duration (hours)^*∗*^	Minimum-maximum daily ambient temperature (°C)	Statistical analysis provided (Y/N)
[34]	Retrospective	M	N	69/31	60	9/7–7/8/2013	17	15–35	Y
						29/6–27/7/2014			

[35]	Retrospective	S	Y	51.5/48.5	49.7	6/6−5/7/2016	15	28–43	Y
				55.5/44.5	50.4	27/5–24/6/2017			

[36]	Prospective	S	N	42/58	45.6	11/8–8/9/2010	15	15–40	Y

[37]	Prospective	S	N	47/53	75	29/6–27/7/2014	17	15–35	Y

[38]	Prospective	S	N	61/39	53.1	18/6–17/7/2015	15	25–45	Y

[39]	Prospective	S	N	50/50	39	NR	NR	NR	Y

[40]	Prospective	S	N	40/60	53	16/11–15/12/2001	12	10–25	Y
				100/0	48.3				

[41]	Prospective	S	N	61/39	54	4/10–3/11/2005	13	20–30	Y

[42]	Prospective	S	N	57/43	44	August/2011	14	22–38	Y
						July–August/2012			

[43]	Prospective	S	Y	17/83	45.6	18/6–17/7/2015	17	17–33	Y
				42/58	47.9				

[44]	Prospective	S	Y	60/40	58.4	22/8–19/9/2009 11/8–8/9/2009 1/8–30/8/2009	15	25–40	Y
				50/50	60.9				

[45]	Prospective	S	Y	70/30	66.8	18/6–17/7/2015	17.5	18–30	Y
				51/49	64.1				

[46]	Prospective	S	Y	54/46	68	7/6–5/7/2016	17	15–40	Y
				47/53	69				

[47]	Prospective	S	Y	51.5/48.5	58.7	16/5–14/6/2018	17	5–30	Y
				49.3/50.7	60.1				

[48]	Prospective	S	Y	64/36	65.4	6/5–3/6/2019	16.5	10–25	Y
				62.5/37.5	61.2				

[49]	Prospective	S	N	56/44	60.5	6/6–5/7/2016	15	30–45	Y

[50]	Prospective	S	N	40/60	61.9	16/5–14/6/2018	16	18–35	Y

[51]	Prospective	S	N	36/64	46	6/5–3/6/2019	14.5	25–45	N

[52]	Prospective	S	N	80/20	48.7	6/6–5/7/2016	16	25–40	Y
				33/67	49.7				
				67/33	49				
[53]	Prospective	S	N	38/62	55.1	6/6–5/7/2016	16	25–40	Y
					55.3				
					47.7				

[54]	Prospective	S	N	54/46	49.9	27/5–24/6/2017	13	24–32	Y
				55/45	54.7				

[55]	Prospective	S	N	47/53	53.1	6/5–3/6/2019	14.5	25–40	Y

[56]	Prospective	S	N	21/79	57.2	6/5–3/6-2019	15.5	14–43	Y

[57]	Prospective	S	N	30/70	50	11/8–8/9/2010	15	25–42	Y

[58]	Prospective	S	N	23/77	52.1	11/8–9/9/2010	14	23–32	Y

M, multicenter; S, single center; Y/N, yes/no; M/F, male/female; NR, not reported. ^*∗*^Dates for the summer months and the corresponding average temperature and average durations are for the northern hemisphere.

**Table 2:** Methods of impact of Ramadan fasting on incidence of nephrolithiasis.

Reference	Type of study	Number of centers	Nonfasting controls (Y/N)	Sex (M/F)	Average age (years)	Dates^*∗*^ (summer: 5/15–9/15)	Fasting duration (hours)	Minimum-maximum daily ambient temperature (°C)	Statistical analysis provided (Y/N)
[59]	Prospective	M	Y	69/31	36.4	NR	NR	NR	Y

[60]	Prospective	S	N	100/0	20–45	NR	NR	NR	Y

[61]	Retrospective	S	N	75/25	45.8	Dec–Feb/1998–2002 June–Aug/2011–2015	12	0–25	Y
							15	26–46	

[62]	Prospective	S	N	100/0	41.7	1/9–30/9/2008	14	34-17	Y

[63]	Retrospective	S	Y	71/29	40	16/5–14/6/2018	14.5	30–46	Y
						6/5–3/6/2019			

[64]	Retrospective	M	N	72/28	37.6	27/11–26/12/2000	13.5	15–35	Y

[65]	Retrospective	S	Y	100/0	NR	Mar-Apr/1992	13.5	15–40	Y
						Feb-Mar/1993			
						Feb-Mar/1994			

[66]	Prospective	S	N	64/36	40.5	16/5-14/6/2018	15.5	23–44	Y

M, multicenter; S, single center; Y/N, yes/no; M/F, male/female; NR, not reported. ^*∗*^Dates for the summer months and the corresponding average temperature and average durations are for the northern hemisphere.

**Table 3:** Methods of studies on impact of Ramadan fasting on dialysis patients.

Reference	Type of study	Number of centers	Nonfasting controls (Y/N)	Sex (M/F)	Average age (years)	Dates^*∗*^ (summer: 5/15–9/15)	Fasting duration (hours)	Minimum-maximum daily ambient temperature (°C)	Statistical analysis provided (Y/N)
[58]	Prospective	S	N	53/47	47.7	11/8–9/9/2010	14	23–32	Y

[67]	Retrospective	S	Y	47/53	60	1989–2012	—	—	Y

[68]	Prospective	M	Y	52/48	53.3	29/6–27/7/2014	14	25–49	N
				56/44	58.4				

[69]	Prospective	S	Y	68/32	56.9	16/5–14/6/2018	16	20–42	Y
				59/41	55.2				
				51/49	59.4				

[70]	Prospective	M	Y	60/40	49.9	6/6–5/7/2016	16	25–40	Y
				54/46	49.5				
				58/42	53.7				

[71]	Prospective	S	Y	65/35	47.7	NR	NR	NR	Y
				52/48	51.6				

[72]	Prospective	M	N	46/54	54	1/8–30/8/2011	14	23–43	Y

[73]	Prospective	M	N	55/48	54.3	16/5–14/6/2018	13.5	25–35	Y

[74]	Prospective	S	N	NR	35.5	NR	NR	NR	Y

[75]	Prospective	S	N	44/56	41.8	22/8–19/9/2009	14	25–45	Y

M, multicenter; S, single center; Y/N, yes/no; M/F, male/female; NR, not reported. ^*∗*^Dates for the summer months and the corresponding average temperature and average durations are for the northern hemisphere.

**Table 4:** Methods of studies on impact of Ramadan fasting on kidney transplantation recipients.

Reference	Type	Number of centers	Nonfasting controls (Y/N)	Sex (M/F)	Average age (years)	Dates^*∗*^ (summer: 5/15 –9/15)	Fasting duration (hours)	Minimum-maximum daily ambient temperature (°C)	Statistical analysis provided (Y/N)
[76]	Prospective	S	Y	58/42 matched?	33.5	15/10–13/11/2004	13.5	10–30	N
					38.3				

[77]	Prospective	S	N	55/45	41.7	1/9–30/9/2008	14	20–39	N
					44.9				

[78]	Prospective	S	N	50/50	39	NR	NR	NR	Y

[79]	Prospective	S	Y	46.5/53.5	43.7	1/8–30/8/2011	14.5	30–49	Y
				57/43	41.8				

[80]	Retrospective	S	Y	64/36	44.2	19/6–27/7/2014	14	25–49	Y
				62/38	43.6				

[81]	Prospective	S	N	64/36	38.7	23/2–23/3/1993	13	15–38	Y
					39.9				

[82]	Prospective	S	N	78/22	35.5	22/1/–19/2/1996	12.5	5–25	Y

[83]	Prospective	S	N	64/36	26.4	10/1–8/2/1997	12.5	0–20	Y

[84]	Prospective	S	Y	63/37	37.3	27/11–26/12/2000	12	10–30	Y
				58/42	40.1				

[85]	Prospective	S	Y	69/31	39.5	Oct-Nov/2004–2006	13	15–35	Y
				76/24	41.5				

[86]	Prospective	S	N	45/55	47	15/10–13/11/2004	13	20–30	Y

[87]	Prospective	M	Y	71/29	42	13/9–12/10/2007	14	20–35	Y
				60/40	43				

M, multicenter; S, single center; Y/N, yes/no; M/F, male/female; NR, not reported. ^*∗*^Dates for the summer months and the corresponding average temperature and average durations are for the northern hemisphere.

**Table 5:** Results of studies on impact of Ramadan fasting on subjects with or without chronic kidney disease.

Reference	Methods	Outcomes
[34]	Retrospective study from Erzincan, Turkey on 117 fasting CKD patients (Stages 2–3) comparing those who developed AKI while fasting (*n* = 27) to those who did not (*n* = 90) during Ramadan in 2013 and 2014	HTN and number of days fasting were significant risk factors for AKI. RAASi were not risk factor. Patients with AKI tended to be older with higher baseline creatinine and proteinuria. Patients were evaluated only during Ramadan. No eGFR reported before, during, or after Ramadan
[35]	Retrospective study from Riyadh, KSA that included 499 patients admitted for AKI during Ramadan compared to 499 patients admitted for AKI during the following lunar month (Shawal) in 2016 and 2017. Ramadan Fasting was assumed	Incidence of AKI during Shawal was significantly higher than during Ramadan. HTN, a history of AKI, and cirrhosis were significant predictors. The higher incidence of AKI in Shawal may be the result of higher ambient temperatures during Shawal compared to Ramadan
[36]	Prospective study from Fez, Morocco on 60 CKD patients divided into three groups:	Seven patients (12%) developed AKI (one at Stage 1, five with Stage 2, and one with Stage 3). Five patients recovered completely and two recovered partially. A baseline Cr Cl <60 mL/min was the only risk factors identified. Mean baseline Cr Cl for all patients was 72.9 mL/min. No Cr Cl values during and after Ramadan were reported
	Group 1: 29 patients with Cr Cl >60 mL/min	
	Group 2: 26 patients with Cr Cl = 30–59 mL/min	
	Group 3: 5 patients with Cr Cl = 15–29 mL/min	
[37]	Prospective study from Konya, Turkey designed to determine the incidence of AKI during Ramadan fasting. The study included 45 fasting subjects with mean eGFR >80 mL/min/1.73 m^2^	AKI Stage 2 was reported immediately before Iftar as per AKIN-urinary output criteria that was accompanied by a small but significant increase in mean creatinine that did not meet AKIN criteria (thirst period <48 hours long and creatinine increase <26.5 *μ*mol/L)
[38]	Prospective study from Riyadh, KSA on 65 CKD-ND (Stage 3–5) patients with mean eGFR 31 mL/min. No eGFR data during or after Ramadan were provided	WKF developed in 34%. WKF developed during in 15 patients and after Ramadan in seven, including 7/36 with Stage 3, 12/24 with Stage 4, and 3/5 with Stage 5. Only eight patients improved. Risk factors included a lower baseline eGFR, higher baseline systolic BP, and younger age. Fasting had no apparent impact on potassium levels
[39]	Prospective study from Dammam, KSA that included 36 fasting CKD patients with Cr Cl <35 mL/min. Dates were not reported	Statistically significant deterioration in Cr Cl from pre-Ramadan average values of 17.2 ± 3.5 mL/min to immediate and two-week post-Ramadan average values of 13.2 ± 2.2 and 13.7 ± 3.2 mL/min, respectively
[40]	Prospective study from Alexandria, Egypt that included 12 CKD patients with a mean eGFR of 33 mL/min but no severe heart or liver disease. The control group included six healthy men	No significant changes in GFR were observed, although urinary NAG tended to rise in those with CKD signaling renal tubular injury. Significantly higher serum potassium levels were observed during Ramadan among those in the CKD group. No significant differences in BP were reported
	GFR was measured with Tc-99m stannous DTPA renography before and during Ramadan	
[41]	Prospective study from Al-Ain, UAE that included 31 patients with CKD-ND (Stages 3–5) with a mean Cr Cl of 29.7 mL/min determined by CG formula. Patients evaluated before, during the final week, and one month after Ramadan	Statistically significant improvements in eGFR during and after Ramadan (30.9 and 32.7 mL/min, respectively) accompanied by nonsignificant drop in BP and weight with no risk of hyperkalemia and no effect on PCR
[42]	Prospective study from Zagazig, Egypt that included 30 fasting patients with DM, 63% with a mean baseline ACR >30	ACR increased significantly from a pre-Ramadan baseline mean of 98.41 ± 160.49 compared to a post-Ramadan mean of 141.49 ± 228.62. No eGFR values were reported
[43]	Prospective study from Istanbul, Turkey on 23 fasting (eGFR 86 mL/min/1.73 m^2^) versus 31 nonfasting (eGFR 66 mL/min/1.73 m^2^) PCKD patients	No significant changes in urine biomarkers (NGAL, KIM1), eGFR, potassium, weight, or 24-hour urine volume in either group. Significant reduction in proteinuria and insignificant reduction in BP were observed in the fasting group
[44]	Prospective study from Cairo, Egypt on 52 fasting CKD patients (eGFR 27.7 mL/min/1.73 m^2^) versus 54 nonfasting CKD patients (eGFR 21.5 mL/min/1.73 m^2^)	Higher risk of CV events in patients with preexisting CVD and an early increase in creatinine levels. Values for eGFR dropped during Ramadan with RAASi and diuretics. Creatinine levels were not significantly different from baseline when evaluated three months after Ramadan
[45]	A prospective study from Rize, Turkey that included 45 fasting CKD-ND (Stage 3–5) patients (42.6) versus 49 nonfasting CKD patients (31.9)	No significant changes in mean eGFR or BP before and after Ramadan were observed between groups, but a trend toward improved mean eGFR values among those in the fasting group. Four patients in each group developed WKF; these individuals were older and treated with diuretics
[46]	A prospective study from Corum, Turkey 2–7/Jun–5/Jul/2016 that included 24 fasting (eGFR 35 mL/min/1.73 m^2^) and 55 nonfasting (eGFR 34 mL/min/1.73 m^2^) CKD patients	No statistically significant differences between the groups were observed with respect to WKF (25% reduction in eGFR or 26.5 *μ*mol/L rise in creatinine levels), which was reported in 12.5% of fasting versus 7.5% nonfasting CKD patients. DM and proteinuria were risk factors for WKF
[47]	A prospective study from London, UK that included 68 fasting (eGFR 47 mL/min/1.73 m^2^) and 71 nonfasting (eGFR 48 mL/min/1.73 m^2^) diabetic CKD patients	No significant changes in weight, BP, creatinine, and urinary PCR evaluated both pre- and post-Ramadan were observed either within or between the two groups. No episodes of AKIs were reported
[48]	A prospective study from Ordu, Turkey included 64 fasting (eGFR 46.2 mL/min/1.73 m^2^) and 66 nonfasting (eGFR 35.8 mL/min/1.73 m^2^) CKD-ND (Stages 3–5) patients	A small but statistically significant increase in eGFR was observed among those in the fasting group (to 48 mL/min/1.73 m^2^) and a corresponding decline (to 33 mL/min/1.73 m^2^) in the nonfasting group. No effects on ACR or serum potassium levels were reported
[49]	A prospective study from Dubai, UAE that included 19 fasting diabetic CKD patients with average baseline eGFRs of 48.9 mL/min/1.73 m^2^	No significant changes in eGFR, weight, BP, or serum potassium levels were reported
[50]	Study from Mansoura, Egypt that included 20 fasting CKD patients with an average baseline eGFR of 25.7 mL/min/1.73 m^2^	No significant changes in creatinine were reported. A nonsignificant rise in eGFR (to 28.3 mL/min/1.73 m^2^) was observed after Ramadan. No significant changes in weight, BMI, muscle and bone mass, total body water, or visceral fat were observed after Ramadan fasting
[51]	Prospective study from Hyderabad, India that included 28 fasting CKD patients	No significant differences in average eGFR for patients before and after Ramadan (56 versus 54.5 mL/min/1.73 m^2^). Four CKD 4 and 5 patients exhibited WKF. Two of these patients improved after Ramadan and two did not. Low eGFRs were identified as the only associated risk factor
[52]	Prospective study from Mansoura, Egypt divided 90 fasting type 2 DM patients into three groups:	Pre- to post-Ramadan values of ACR increased significantly (71 to 112 and 16 to 42) and eGFR decreased significantly (114 to 78 mL/min/1.73 m^2^ and 113 to 98 mL/min/1.73 m^2^) among those in Groups 2 and 3
	Group 1: ACR >30 and eGFR 60–<90 mL/min	
	Group 2: ACR >30 and eGFR >90 mL/min	
	Group 3: No CKD	
[53]	Prospective study from Fayoum, Egypt that included 337 fasting patients with type 2 DM and a mean pre-Ramadan baseline creatinine levels of ≤88 *μ*mol/L and ACR of ≤80	A small but statistically significant increase in both mean Cr ≤95 *μ*mol/L and mean ACR ≤90 after Ramadan was observed together with a statistically significant reduction in systolic BP
[54]	Prospective study from Singapore that included 68 fasting patients with type 2 DM with eGFR ≥45 mL/min. These patients were divided into groups based on SGLT2i use (yes/no). Patients' mean eGFRs was 88 mL/min/1.73 m^2^ and 70% had an ACR >30	Statistically significant reductions in weight, BP, and baseline eGFR were reported in both groups during Ramadan. Increases in serum potassium levels observed during Ramadan correlated with reductions in eGFR and not with RAASi use
[55]	Prospective, longitudinal study from Karachi, Pakistan that included 70 fasting patients with type 2 DM and mean baseline eGFRs of 75 mL/min. The number of patients with eGFRs <60 mL/min was unknown	The mean eGFR measured six weeks post-Ramadan was significantly reduced (to 63 mL/min/1.73 m^2^). However, 29 patients who presented with a mean baseline eGFR of 72 mL/min experienced no change in mean eGFR when evaluated 12 months later. Values for eGFR dropped equally in CKD and non-CKD patients
[56]	Prospective study from Marrakech, Morocco that included 39 patients with type 2 DM and baseline eGFRs of 130 mL/min; 87% of these patients presented with an ACR <30. It reported changes in mean eGFR and hydration status measured by bioelectrical impedance analysis (BIA)	Significant decline in eGFR to 117 mL/min accompanied by an increase in hydration measured by BIA from 34.7 to 35. Creatinine increased in 67% of patients. Small but significant reductions in BMI, but no comment on BP or ACR. Risk of AKI was significantly higher in patients with baseline eGFRs <60 mL/min/1.73 m^2^, although it is not clear how many of these patients were included in the study
[57]	Prospective study from Banha, Egypt that included 20 healthy subjects with normal Cr Cl (124 mL/min by CG formula) and ACRs of 13 compared to 20 type 2 DM patients (creatinine clearance of 107 mL/min and baseline ACR of 36) who were or were not treated with vitamin E	Significant increases in Cr Cl in the nondiabetic patients treated with vitamin E during Ramadan were observed together with insignificant reductions in ACR throughout. The precise creatinine clearance values were not provided
[58]	Prospective study from Riyadh, KSA that included 39 CKD-ND (Stage 3–5) patients with an average baseline Cr Cl of 40.8 mL/min	No significant changes in eGFR, urine volume, or proteinuria were observed

CKD, chronic kidney disease; HTN, hypertension; AKI, acute kidney injury; RAASi, renin-angiotensin-aldosterone inhibitor; eGFR, estimated glomerular filtration rate; KSA, Kingdom of Saudi Arabia; Cr Cl, creatinine clearance; AKIN, acute kidney injury network; ND, nondialysis; WKF, worsening kidney function; BP, blood pressure; NAG, N-acetyl-*β*-D-glucosaminidase; UAE, United Arab Emirates; CG, Cockcroft-Gault; PCR, protein-creatinine ratio; DM, diabetes mellitus; ACR, albumin-creatinine ratio; PCKD, polycystic kidney disease; NGAL, neutrophil gelatinase-associated lipocalin; KIM1, kidney injury molecule-1; CV, cardiovascular; CVD, cardiovascular disease; SGLT2i, sodium-glucose transporter 2 inhibitor.

**Table 6:** Results of studies on impact of Ramadan fasting on incidence of nephrolithiasis.

Reference	Methods	Outcomes
[59]	Prospective study from Tehran, Iran, that evaluated 24-hour urine volume and total calcium, phosphorous, and magnesium excretion in 57 fasting men, including 37 with a history of calcium calculi	Urine calcium was significantly lower and urine uric acid, citrate, phosphorus, sodium, and potassium were significantly higher during fasting compared to nonfasting state. Uric acid supersaturation increased, and calcium phosphate supersaturation decreased significantly during fasting. No significant increase in calcium oxalate supersaturation or risk of calculus formation during fasting
[60]	Single-center prospective study from Kuala Lumpur, Malaysia, (date not reported) that evaluated 24-hour urine samples collected from 20 healthy fasting men before, weekly during, and one to four weeks after Ramadan	No significant changes in 24-hour urine volume were observed. Lower 24-hour urine sodium levels, higher urine osmolality, and decreased urine titratable acidity were reported. No protein, glucose, urobilinogen, ketones, or hemoglobin were detected
[61]	Retrospective cross-sectional study from Riyadh, KSA, that included 237 consecutive patients presenting to an emergency department with renal colic only during Ramadan in 1998–2002 (Dec–Feb) and 2011–2015 (Jun–Aug). The incidence of renal colic during Ramadan in the five years in which it occurred in winter *versus* summer months was compared to the incidence during both summers and winters that did not include Ramadan	Fasting was not confirmed. Nephrolithiasis was confirmed by computed tomography. Thirty-seven percent of the patients were male. The risk of developing urinary stones during Ramadan was similar to the risk observed in nonfasting months. However, Ramadan fasting during the summer may increase the risk of stones in the ureters compared to those presenting at other locations when compared to Ramadan fasting during the winter months
[62]	Prospective study from Mashhad, Iran that included 610 patients admitted for renal colic who had been diagnosed clinically two weeks before, during, and two weeks after Ramadan (i.e., between Aug and Oct of 2008)	The incidence of renal colic was highest during the first two weeks of Ramadan when compared with the other periods evaluated. However, incidence declined during the last two weeks of Ramadan; this decline continued after Ramadan. The authors ruled out ambient temperature as a risk factor. Values for eGFR were not reported
[63]	Retrospective study from Manama, Bahrain that included 809 Muslims admitted with renal colic during the month before, the month of, and the month after Ramadan over a two-year period (2018 and 2019). Renal colic was diagnosed clinically (not radiologically). Fasting was assumed	The number of admissions during Ramadan (38.2%) and the month following (37.1%) was significantly higher than the number of cases in the month just before Ramadan (24.7%). The impact of temperature differences between the three periods was not evaluated. Kidney function was not reported
[64]	Multicenter retrospective study from Varamin, Iran, that included 574 subjects admitted with a diagnosis of renal colic from Mar 1, 2000, to Mar 1, 2001. Incidence over the 12-month period was reported	Number of hospital admissions for renal colic during Ramadan was not significantly different from mean number of admissions for renal colic during any of the other months of the lunar year. Mean admissions during the warmer seasons were significantly higher than during Ramadan
[65]	Single-center retrospective study from Jeddah, KSA, that included male patients seen in the emergency department for renal colic and who were diagnosed clinically one month before, during, and one month after Ramadan over three years (Mar-Apr/1992, Feb-Mar/1993, and Feb-Mar/1994)	Significant correlation with ambient temperature but not with fasting (which took place during the cooler months) or humidity. Fasting status and kidney function were not reported
[66]	Prospective single-center study from Lahore, Pakistan, that reported the incidence of clinically-diagnosed renal colic	A similar number of emergency department visits were reported during the months of Ramadan and Shawal despite the warmer weather during Ramadan

KSA, Kingdom of Saudi Arabia; eGFR: estimated glomerular filtration rate.

**Table 7:** Results of studies on impact of Ramadan fasting on dialysis patients.

Reference	Methods	Outcomes
[58]	Prospective study from Riyadh, KSA, that included 32 fasting HD patients	No change in Kt/V, although creatinine increased and 25% of patients developed hyperkalemia. Despite the high frequency of fluid overload (>4% IDWG) accompanied by elevated BP, no hospitalization was needed
[67]	Retrospective analysis from Karachi, Pakistan, that evaluated mortality rates during Ramadan (1989–2012) in a cohort of 1,841 HD patients. Fasting status was unknown	Forty-nine percent of HD patients died during the study period. The highest rates of death in the Hijri calendar were observed during Ramadan (10.5% of the mortality) but this was attributed to dietary changes and/or cooler weather. Of note, the highest rates of death in the Gregorian calendar were in the cooler months of December and January
[68]	Prospective study from Riyadh, KSA, that included 407 fasting and 228 nonfasting HD patients	Increased number of missing sessions for fasting patients only. IDWG and pre and postdialysis BP were slightly but not significantly higher in the fasting group. No differences in serum potassium were observed during Ramadan
[60]	Prospective study from Nablus, Palestine, that divided HD patients into three groups:	The authors reported a higher mean IDWG among those participating in daily fasting and a slightly higher mean IDWG for those engaged in partial fasting (especially those with DM). Patients participating in daily fasting had higher serum potassium levels
	Group 1: daily fasting (*n* = 31)	
	Group 2: fasting on non-HD days (*n* = 102)	
	Group 3: nonfasting (*n* = 136)	
[70]	Prospective study from Mansura, Egypt, that included HD patients divided into three groups:	Younger patients with fewer comorbidities in the fasting groups exhibited lower mortality rates than older and sicker patients who were not fasting. All groups showed small but statistically significant drops in BP. Higher albumin levels were detected in fasting groups. Episodes of hypotension and hypoglycemia were more frequent in the fasting group
	Group 1: daily fasting (*n* = 381)	
	Group 2: fasting on non-HD days (*n* = 574)	
	Group 3: nonfasting (*n* = 1090)	
[71]	Prospective study from Karachi, Pakistan, that included 34 fasting and 252 nonfasting HD patients. Dates were not reported	Fasting resulted in a significant drop in diastolic BP only and an increase in albumin. No significant elevations in weight or serum potassium levels were observed
[72]	Prospective study from Kuala Lumpur, Malaysia, that included 35 fasting HD patients	Significant drop in predialysis weight gain that was even more prominent among nondiabetics. No significant changes in dry weight, IDWG, or serum potassium were observed. Significant increases in albumin in all groups, along with a significant drop in Hgb levels in diabetics
[73]	Prospective study from Klang Valley, Malaysia, that included 87 fasting HD patients (22% engaged in partial fasting)	Significant decreases in IDWG and waist circumference were observed in response to regular Ramadan fasting, especially among females. Significant decreases in phosphorus, albumin, urea, and creatinine were observed. No significant changes in diet were reported
[74]	Prospective study from Riyadh, KSA, that included 40 HD patients who were fasting on-off HD days. Clinical and laboratory parameters were compared to pre-Ramadan values. The study dates were not reported	Small but significant increases in IDWG and serum potassium levels were observed during Ramadan compared to pre-Ramadan, but no emergencies developed. No significant changes in BP were observed
[75]	Prospective study from Riyadh, KSA, that included fasting PD patients (8 CAPD and 10 CCPD) without a control group. A pre-Ramadan evaluation was performed to rule out any contraindications to fasting	Nutritional counseling was provided to ensure adherence to appropriate fluid, salt, potassium, and calorie intake to avoid dialysis emergencies. No changes in weight, urine output or Cr Cl were observed, nor were there any serious event

KSA, Kingdom of Saudi Arabia; HD, hemodialysis, IDWG, interdialytic weight gain; BP, blood pressure; Hgb, hemoglobin; PD, peritoneal dialysis; CCPD, continuous cycler peritoneal dialysis; CAPD, continuous ambulatory peritoneal dialysis.

**Table 8:** Results of studies on the impact of Ramadan fasting on kidney transplantation recipients.

Reference	Methods	Outcomes
[76]	Prospective study from Tehran, Iran, that included 19 fasting and 20 nonfasting kidney transplant recipients	No significant change in serum creatinine from a baseline level of <135 *μ*mol/L
[77]	Prospective study from Tripoli, Libya, that included 20 fasting (Cr Cl 96 mL/min for M and 101 mL/min for F) kidney transplant recipients	All completed the fast
		Nonsignificant increase in Cr Cl as well as no change in weight, BP, or urine volume
[78]	Prospective study from Tabriz, Iran, that included 24 kidney transplant recipients with creatinine levels <160 *μ*mol/L	No changes in serum Cr, electrolytes, BP, or urine volume were observed. Study dates were not reported
[79]	Prospective study from Riyadh, KSA, that included 43 fasting (eGFR 75.6 mL/min/1.73 m^2^) and 37 nonfasting (eGFR 65.9 mL/min/1.73 m^2^) kidney transplant recipients	Mean and percentage changes in eGFR were similar in the two groups. Mean eGFR values were similar pre- and post-Ramadan in the fasting group regardless of baseline eGFR. No increased risks of rejection, UTI, or calculi reported
[80]	Retrospective study from Riyadh, KSA, that included 280 fasting (eGFR 72.7 mL/min/1.73 m^2^) and 285 nonfasting (eGFR 72.8 mL/min/1.73 m^2^) kidney transplant recipients	No statistically significant differences in eGFR, BP, proteinuria, or drug levels were observed after compared to before Ramadan. Baseline eGFR and baseline proteinuria were statistically significant risk factors for a change in eGFR as determined by regression analysis
[81]	Prospective study from Madina, KSA, that included 11 recipients of a kidney transplant more than one year previously who were maintained on steroids, azathioprine, and cyclosporine	No significant changes in kidney function (nb: eGFR and Cr Cl not recorded). Serum potassium increased during Ramadan but remained within normal range. The observed decrease in FENa did not achieve statistical significance
[82]	Prospective study from Riyadh, KSA, that included 23 kidney transplant recipients with a baseline creatinine of 117 *μ*mol/L	No significant change in Cr or serum/urine electrolytes in patients with normal or low eGFRs except for an observed increase in serum potassium levels (albeit remaining within the normal range) in patients with normal eGFR
[83]	Prospective study from Algiers, Algeria, that included 14 participants who received a kidney transplant less than a year before the study and presented with a mean baseline creatinine of 115 and normal BP	No significant change in BP, weight, Cr levels, or urine volume was observed. The authors reported a significant increase in uric acid, urea, protein, and urinary levels of sodium and potassium, and a significant drop in proteinuria
[84]	Prospective study from Kuwait that included 71 fasting and 74 nonfasting kidney transplant recipients. Baseline creatinine levels were 98 and 103 for those who were fasting and nonfasting, respectively	Participants were matched for age, sex, BMI, and posttransplant time. Participants were followed for 12 months after Ramadan. No significant changes in Cr or BP were observed and there was no significant difference in the incidence of adverse events
[85]	Prospective study from Riyadh, KSA, that included 35 fasting and 33 nonfasting kidney transplant recipients. Mean eGFR in the nonfasting group was lower than that of the fasting group; 63% presented with an eGFR <60 mL/min/1.73 m^2^	Comparable results were obtained with respect to sex (mainly M), age, DM status, time from transplant, and immunosuppressives. Values for eGFR did not differ significantly from baseline in the fasting group or between groups. No differences in MAP or UP were observed
[86]	Prospective study from Al-Ain, UAE, that included 22 kidney transplant recipients with a mean eGFR of 68.2 mL/min/1.73 m^2^	No change in eGFR, BP, weight, or potassium levels
[87]	Prospective study from Tehran, Iran, that included 41 fasting (eGFR 73 mL/min/1.73 m^2^) and 41 nonfasting (eGFR 73 mL/min/1.73 m^2^) kidney transplant recipients matched for age, sex, BMI, donor source, immunosuppressive therapy, and transplant duration	No changes in eGFR or BP were observed. No acute complications developed. Sixteen patients with eGFR 28–59 mL/min/1.73 m^2^ exhibited no change in eGFR. Previous repeated fasting led to an increase in eGFR but no significant effect attributed to fasting

BP, blood pressure; Cr Cl, creatinine clearance; M, males; F, females; KSA, Kingdom of Saudi Arabia; eGFR, estimated glomerular filtration rate; UTI, urinary tract infection; FENa, fractional excretion of sodium; BMI, body mass index; MAP, mean arterial pressure; UP, urinary protein; UAE, United Arab Emirates.

## Data Availability

Previously published studies were used to support this review. These prior studies (and datasets) are cited at relevant places within the text as references [34–87].
